# Perianal Crohn’s disease and hidradenitis suppurativa: a possible common immunological scenario

**DOI:** 10.1186/s12948-015-0018-8

**Published:** 2015-07-22

**Authors:** Francesco Giudici, Laura Maggi, Raffaella Santi, Lorenzo Cosmi, Francesco Annunziato, Gabriella Nesi, Giusi Barra, Gabrio Bassotti, Raffaele De Palma, Francesco Tonelli

**Affiliations:** Department of Surgery and Translational Medicine, University of Florence, Largo Brambilla 3, 50139 Florence, Italy; Department of Experimental and Clinical Medicine, University of Florence, Florence, Italy; Department of Clinical and Experimental Medicine, Second University of Naples, Naples, Italy; Department of Medicine, University of Perugia, Perugia, Italy

**Keywords:** Hidradenitis suppurativa, Crohn’s disease, T helper lymphocytes

## Abstract

**Background:**

Crohn’s disease (CD) and Hidradenitis suppurativa (HS) are both chronic inflammatory diseases. The pathogenesis of these diseases is multifactorial, due to the interaction of genetic and environmental factors leading to a deregulated local immune response where T lymphocytes play a major role. To the best of our knowledge, no previous study has clarified whether the pathogenetic mechanism of perianal CD and HS is the same. We therefore analyzed the cellular expression pattern and the cytokine repertoire in three patients suffering from both perianal CD and HS.

**Methods:**

We evaluated three patients affected by concurrent HS and CD with fistulizing perianal disease. Surgical specimens have been fixed and embedded in paraffin prior to sectioning for histological examination. Inflammatory tissue curettages have been recovered during intervention from perianal fistulas and HS lesions in order to analyze the phenotypic and functional characteristics of infiltrating T cells. In particular we evaluated T cells, by flow cytometry, for cytokine production profile and expression of surface markers. Moreover, analysis of the T cell repertoire was performed by means of spectratyping, in only one patient.

**Results:**

A higher frequency of CD4+ CD161+ T lymphocytes has been detected in CD fistulas and in HS lesions than in peripheral blood (PB) samples. In the patient in whom we derived enough cells from the three sources, we found higher frequency of CD4+ IL-17- producing cells in HS lesion and fistula lesion compared to PB. It is noteworthy that the same clonotypes were expanded in this patient in T cells derived from both HS lesion and fistula lesion.

**Conclusion:**

The presence of numerous CD4+ CD161+ lymphocytes in fistula and HS lesion curettages suggests that these cells may play a pathogenic role, and candidates CD161 as a possible biological target for medical treatment.

## Background

Crohn’s disease (CD) and hidradenitis suppurativa (HS) are chronic inflammatory diseases. HS involves the hair follicles in apocrine glandular zones, affecting the axillae, groins, external genitals or perianal regions. It is characterized by recurrent, spontaneously draining, suppurative lesions and long-lasting subcutaneous sinus tracts, cutaneous fistulae or dermal-cutaneous scars [1].

The prevalence of HS is approximately 1 in 300 and typically commences during puberty and the teenage years, more frequently in females. Familial history is documented in a quarter of the patients with an autosomal inheritance [1].

CD may involve any part of the gastrointestinal tract and is defined by the presence of discontinuous, transmural, inflammatory lesions [2]. Abdominal pain and weight loss are the typical presenting symptoms. CD can be divided into three forms: inflammatory, fistulizing or obstructive. Between 60% and 80% of CD patients suffer from perianal fistulas [3,4]. Previous reports have suggested a possible association between CD and HS (Table 1) [5-16].

**Table 1:** **Association between CD and HS as reported in the literature (HS-CD = diagnosis of HS predated that of CD; CD-HS = diagnosis of CD predated that of HS)**

**Author**	**Year**	**N° patients**	**Sex**	**Sequence**
**LS Ostlere**	1991	3	F,F,M	CD-HS, CD-HS, CD-HS
**C Gower-Rousseau**	1992	3	M	CD-HS, CD-HS, CD-HS
**NP Burrows**	1992	2	M,F	HS-CD, HS-CD
**RL Attanoos**	1993	3	F,M,M	CD-HS, HS-CD, CD-HS
**AA Kafity**	1993	1	M	CD-HS
**JM Church**	1993	61	Both	Multiple
**EV Tsianos**	1995	1	M	CD-HS
**MK Roy**	1997	1	M	HS-CD
**F Martinez**	2001	1	F	CD-HS
**M Roussomoustakaki**	2003	1	F	HS-CD
**HH Van der Zee**	2009	102	Both	Multiple
**S Yazdanyar**	2010	2	F,F	HS-CD, HS-CD

Although smoking is regarded as a risk factor of both CD and HS recurrence, the etiology of these diseases is still largely unknown and is likely to be multifactorial. It is hypothesized that genetic and environmental factors may cooperate in leading to a deregulated local immune response where T lymphocytes play a major role [1,17]. CD4+ T helper (Th) cells can be divided into different subsets according to their cytokine production profile: Th1 cells produce interferon (IFN)-γ, thus protecting against intracellular pathogens, Th2 cells produce interleukins (IL)-4, IL-5, IL-9 and IL-13 and offer defence against parasitic helminths, while Th17 cells produce IL-17 and protect from fungal infections [18]. In addition to their protective role against pathogens, Th lymphocytes also contribute to the development of immune-mediated disorders: in particular both Th1 and Th17 cells have been credited to being involved in inducing and maintaining an intestinal inflammatory reaction in experimental animal models as well as in human CD [18,19]. Furthermore, biological therapy targeting tumor necrosis factor (TNF)-α with specific monoclonal antibodies, has shown to be particularly effective in both CD and HS treatment [1,6,17]. Recently, local injection of anti-TNF-α antibodies (Ab) has proved to be a safe and effective therapy for perianal fistulas in CD [4,20].

We recently observed three CD patients with perianal fistulas and suffering from HS, who gave us the opportunity to compare the cellular and cytokine expression profile of these lesions.

## Methods

Between February and October 2011, we operated three patients affected by concurrent HS and CD with fistulizing perianal disease. Surgical treatment consisted in resection of the HS lesion, followed by fistulotomy (n = 1) or fistulectomy (n = 2). Surgical specimens were fixed in 10% neutral buffered formalin and embedded in paraffin prior to sectioning for histological examination. Inflammatory tissue curettages were recovered during intervention from perianal fistulas and HS lesions in order to analyze the phenotypic and functional characteristics of the infiltrating T cells. These features were then compared with those of peripheral blood T lymphocytes.

To isolate T lymphocytes, tissue fragments were mechanically disrupted with a MEDI machine (Becton Dickinson). Fluorochrome-conjugated mAbs and isotype-matched control mAbs were purchased from BD Biosciences (San Jose, CA). The fluorochrome-conjugated anti-IL-17 mAbs was obtained from eBioscience (San Diego, CA). PMA, ionomycin and brefeldin A were purchased from Sigma Chemical Co. (St. Louis, MO). Mononuclear cells (MNCs) from the different tissues were analyzed for intracellular cytokine production as previously described [18]. All procedures in the study are in accordance with the ethical standards of the Regional Committee on Human Experimentation.

Analysis of the T cell repertoire was performed by means of spectratyping, a PCR-based technique which allows to assess CDR3 region length in the TCR families. The CDR3 region is unique for a given T cell and consequently all cells bearing a particular CDR3 will belong to that clone. Due to an allelic exclusion phenomenon, only a TCR-BV gene is predictively rearranged in each T cell. Therefore, according to TCR-BV gene, modification of specific clonotypes can be monitored both *in vivo* and *in vitro*. With this technique, a TCR family is graphically described by a series of peaks with Gaussian distribution. Alteration of intensity and/or distribution of the peaks indicates a perturbation of the T cell repertoire. Extended peaks are directly sequenced in order to assess the clonotype composition of each peak [21].

## Results

The three patients were Caucasian females, two of them were smokers. CD has been diagnosed at a mean age of 23 years and histologically confirmed in all patients. CD was ileal in 2 patients and colic in one; it was stricturing in all three. Perianal fistulas have occurred at a mean age of 26.3 years. In two cases, the fistula was recurrent, trans-sphincteric in two patients and anovaginal in one. HS was diagnosed with a mean of 10.7 years after CD diagnosis. All patients have been medically treated for CD with cycles of steroids and mesalamine, whilst two patients have been also treated with azatioprine and infliximab for a mean of 7 and 8 months, respectively. In all cases immunosuppressive treatment has been suspended one month prior to surgery. The HS lesions were LC1 and Hurley stage II [22,23] in all patients and were localized at the axilla in 2 cases and at the right inguinal region in the remaining case. Table 2 summarizes clinical features of the recruited patients.

**Table 2:** **Clinical features of enrolled patients**

**Patient**	**Sex/age at surgery (years)**	**Smoker**	**Age at CD diagnosis (years)**	**Age at perianal fistula diagnosis (years)**	**CD localization/type of perianal fistula**	**Age at HS diagnosis (years)**	**Type of HS**	**Previous therapy**
1	F/49	Yes	21	20	Ileal/transsphincteric	33	Inguinal, recurrent	Steroid
								Mesalamine
								Azatioprine
								Infliximab
2	F/26	Yes	22	26	Colic/Ano-vaginal	26	Axillary	Steroid
								Mesalamine
								Adalimumab
3	F/42	No	26	33	Ileal/transsphincteric	40	Axillary	Steroid
								Mesalamine
								Azatioprine
								Infliximab

**Figure 1:**
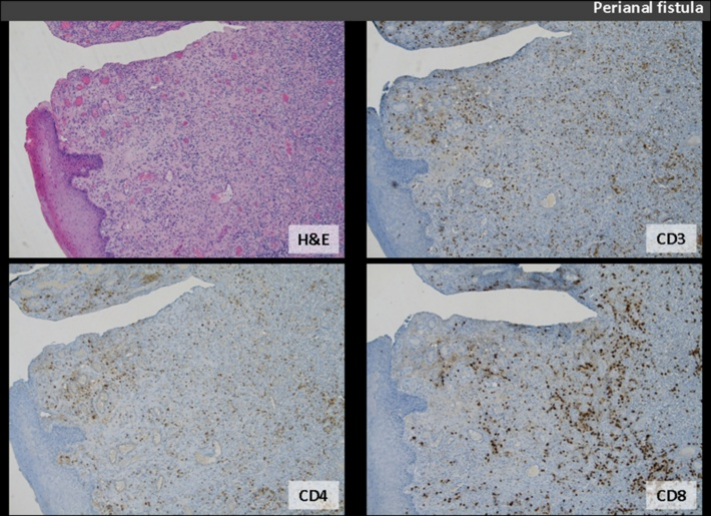
Perianal fistula sample: haematoxylin-eosin (H&E) staining reveals denuded surface, overlying florid inflammatory granulation tissue (original magnification ×5). The CD3+, CD4+ and CD8+ T cells are identified by appropriate immunohistochemical stains (patient n°3).

**Figure 2:**
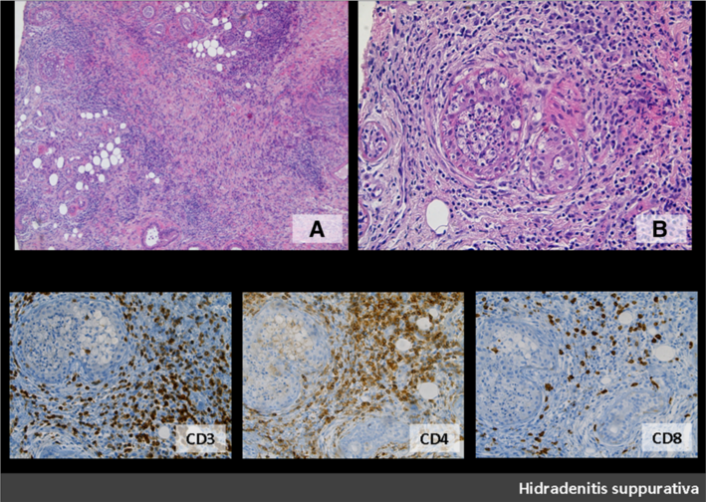
HS sample: in HS, marked acute and chronic inflammatory infiltrate involves apocrine glands as well as adjacent connective tissue (**A**: H&E, original magnification, ×5; **B**: H&E, original magnification, ×20) (patient n°3). Chronic inflammatory infiltrate includes T lymphocytes (CD3+) with CD4+ T cells representing the predominant subtype (original magnification, ×20). (inferior panel: CD3, CD4, CD8).

Histological examination reveals florid inflammatory granulation tissue in perianal CD (Figure 1), and marked inflammatory infiltrate in apocrine glands as well as adjacent connective tissue in HS, the CD4+ T cells representing the predominant subtype in this latter (Figure 2).

**Figure 3:**
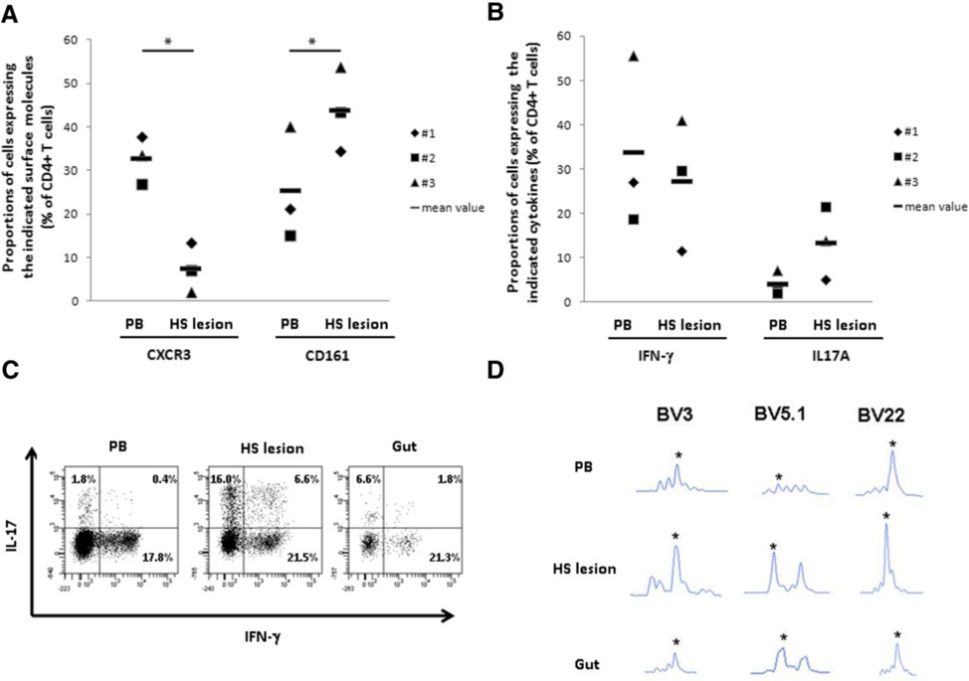
CD4+ CD161+ T cells producing IL-17 accumulate in HS lesion and gut. **(A)** CXCR3 and CD161 expression in CD4+ T lymphocytes obtained from PB and HS lesion of the three patients with HS associated with CD. Symbols represents each single patient. *p < 0.05. **(B)** Cytokines production profile of CD4+ T lymphocytes obtained from PB and HS lesions of the three patients with HS associated with CD. Symbols represents each single patient. **(C)** Flow cytometric plots intracellular staining of IL-17 and IFN-γ cytokines of CD4+ T lymphocytes after *in vitro* polyclonal stimulation, obtained from indicated tissues. One donor is represented. **(D)** Three representative TCR-BV families (TCR-BV3; TCR-BV5.1; TCR-BV22) in HS lesion zones, blood and gut of one patient with HS associated to CD. Asterisks indicate the presence of clonotypes found expanded in the peaks of interest.

MNCs have been isolated from peripheral blood (PB) samples, fistula curettage and HS lesions, and analyzed for surface marker expression and for the ability to produce IL-17 and IFN-γ after polyclonal stimulation. In particular, due to the low number of cells recovered from fistula curettage, analysis of surface markers and intracellular cytokines was possible in all three patients only on PB- and HS lesion-derived cells. The expression of the Th1 associated molecule CXCR3 was significantly lower in HS-derived CD4+ T lymphocytes than in the PB-derived ones. On the contrary, the expression of the Th17 associated molecule CD161 was significantly higher in HS lesion-derived CD4+ T lymphocytes than in those obtained from PB (Figure 3A). Accordingly, CD4+ T lymphocytes producing IFN-γ were more abundant in PB than in HS lesions, whereas CD4+ T cells producing IL-17 were more abundant in HS lesions than in PB (Figure 3B and C). In the only case in which we had the possibility to analyze the three different sources (PB, HS lesion, and fistula curretage), we found more CD4+ IL-17- producing cells in HS lesion and fistula lesion than in PB, even if the CD4+ IFN-γ - producing cells did not differ among the different sources (Figure 3C). It is noteworthy that CD4+ T lymphocytes derived from PB, HS and fistula, in the same patient, exhibited a similar TCRVB distribution suggesting an expansion of the same clonotypes (Figure 3D).

After a follow-up of 24 months one patient had no evidence of disease (patient number 3), whereas in the remaining two patients the HS lesion recurred after 13.2 (patient number 1) and 16.4 months (patient number 2) respectively.

## Discussion

The *primum movens* of HS seems to be related to the occlusion of the hair follicles by keratinous plugging followed by their dilatation and disruption. Secondary bacterial infection of the apocrine glands may result in abscess formation, gland rupture, inflammation and fistula forming. Long-standing HS in the anogenital region has been suggested to precede squamous cell carcinoma [24]. The present study describes the co-morbidity of perianal CD and HS found in three patients. The association between HS and CD has recently been reinforced in nine case reports and two retrospective studies [5-16]. On interviewing 102 CD patients, Van der Zee et al. found a history of recurrent painful boils in the axillae and/or groins compatible with HS in 17% of cases [15]. Church et al. reviewed hospital records of 61 HS patients and observed that in 24 had also been diagnosed for CD [10]. Anal localization of HS may clinically overlap perianal CD with suppurative complications leading to misinterpretation. Noteworthy, HS can arise either before or after CD. In the latest report, CD diagnosis predated that of HS by an average of 3.5 years [10]. In line with these data, in our patients the diagnosis of HS followed that of CD.

Even if performed on a low number of patients, the present study is the first to compare the inflammatory scenario of both perianal CD fistulas and HS lesions. We documented the accumulation of CD161+ T lymphocytes, that are well known to be enriched in the Th17, Th17/Th1 and non classic Th1 phenotypes [20,21], in the CD fistula and HS lesion curettages. Interestingly, Th1 and Th17 lymphocytes have been claimed to play a role in CD pathogenesis [25]. The coexistence of Th1 and Th17 cells in the same microenvironment and their cooperation in the pathogenesis of several inflammatory diseases have also been reported [18,21]. It has also been shown that Th17 cells can shift towards the Th1 phenotype in the presence of IL-12, through an intermediate state in which the cells are capable of producing both IL-17A and IFN-γ (Th17/Th1 subset) [18,21]. Since IL-12 is highly expressed in CD tissue samples [25], we speculate that Th17 cells could be modulated to become Th17/Th1, or even Th1, during the course of the disease. Accordingly, it has recently been suggested that Th1 and Th17 cytokines can act synergistically in the development of the disease [25]. Indeed, some reports describe the effectiveness of the anti-IL-12/23 p40 chain monoclonal antibody ustekinumab, which inhibits receptor-binding of both IL-12 and IL-23, thus blocking Th1 and Th17 differentiation, in the treatment of CD [26]. Moreover it has been recently described the involvement of IL-23/Th17 pathway in the pathogenesis of skin hidradenitis suppurative [27].

The finding of a possible pathogenic association between CD and HS, could have also therapeutical implications. Indeed, treatment with biologics blocking TNF-α has been observed in both diseases [13,14,17], and a favourable outcome has been reported particularly in the early stages of HS. However, refractory cases to anti- TNF-α therapy have also been described as well as patients developing HS under anti-TNF-α treatment [28].

Finally, here we describe a possible association between CD and HS, based on immunological analysis of infiltrating T cells compared to PB; in any case additional factors could be involved in the pathogenesis of the diseases and of course additional studies are necessary to define if other molecules associated to these disease will be common to both or specific of each one [29-31].

## Conclusion

In conclusion, this work reinforces the link between HS and CD, providing evidence that CD4+ CD161+ T lymphocytes accumulate in perianal fistulas and HS in CD patients and may play a crucial role in the pathogenesis of both diseases. Potential therapeutic implications of these results need to be further investigated.

## Abbreviations

CD: Crohn’s disease
HS: Hidradenitis suppurativa
Th: T helper
PB: Peripheral blood
IFN: Interferon
IL: Interleukins
TNF: Tumor necrosis factor
mAbs: Monoclonal antibodies
MNCs: Mononuclear cells
PCR: Polymerase chain reaction
CDR3: Complementarity determining region 3
TCR-BV: T-cell receptor beta-chain variable
